# Cholestasis-Associated Pruritus and Its Pruritogens

**DOI:** 10.3389/fmed.2021.639674

**Published:** 2021-03-09

**Authors:** Jacqueline A. G. M. Langedijk, Ulrich H. Beuers, Ronald P. J. Oude Elferink

**Affiliations:** Amsterdam University Medical Centers, Tytgat Institute for Liver and Intestinal Research, Research Institute Amsterdam Gastroenterology, Endocrinology and Metabolism (AGEM), University of Amsterdam, Amsterdam, Netherlands

**Keywords:** cholestasis, bile formation, cholestasis-associated pruritus, itch, autotaxin, pruritogen

## Abstract

Pruritus is a debilitating symptom of various cholestatic disorders, including primary biliary cholangitis (PBC), primary sclerosing cholangitis (PSC) and inherited progressive familial intrahepatic cholestasis (PFIC). The molecular mechanisms leading to cholestasis-associated pruritus are still unresolved and the involved pruritogens are indecisive. As a consequence of pruritus, patients suffer from sleep deprivation, loss of daytime concentration, auto-mutilation and sometimes even suicidal ideations. Current guideline-approved therapy of cholestasis-associated pruritus includes stepwise administration of several medications, which may alleviate complaints in some, but not all affected patients. Therefore, also experimental therapeutic approaches are required to improve patients' quality of life. This article reviews the current state of research on pruritogens and their receptors, and shortly discusses the most recent experimental therapies.

## Introduction

Cholestasis is the term for diminished or impaired bile flow generated by hepatocytes and cholangiocytes. The cause of cholestasis can be intra- or extrahepatic, and can be genetic or the consequence of an inflammatory or malignant hepatobiliary disease. Next to fatigue, pruritus is the most frequent symptom in patients with chronic cholestatic disorders and may affect more than half of patients with fibrosing cholangiopathies such as primary biliary cholangitis (PBC) or primary sclerosing cholangitis (PSC) at least transiently during their disease course. Depending on the cause of cholestasis, 30–90% of patients suffer from chronic pruritus, which is unresponsive to antihistamines. Chronic pruritus can lead to loss of concentration, sleep deprivation and auto-mutilation or prurigo nodularis due to scratching (1). In most serious cases, suicidal ideations may occur and the burden of pruritus can become the primary indication for liver transplantation. Cholestasis-associated pruritus shows a diurnal rhythm with increased intensity in the late evening and early night. The itch is typically localized at the limbs and soles of the feet and at the forearms and palms of the hands, but may also be generalized.

Liver diseases over a wide range of prevalences are associated with chronic pruritus including PBC, PSC and secondary sclerosing cholangitis (SSC), cholangiocarcinoma, viral hepatitis, drug-induced cholestasis, sarcoidosis hepatis, and rare inherited forms of cholestasis. For intrahepatic cholestasis of pregnancy (ICP) pruritus is one of the mandatory diagnostic criteria and is observed in 0.5–2.0% of pregnant women, PBC is found in about 0.1% of women at an age of 50 years, whereas Alagille syndrome as an example of rare genetic diseases is found in only one of 100,000 children and adolescents. In some cases, such as PBC, pruritus can be the first symptom of the disease. Since this is a relatively rare disease, diagnosis by general practitioners or dermatologists may be difficult.

It has not been established whether environmental, geographical or life-style factors influence the prevalence or severity of cholestasis-associated itch. However, for ICP there are indications for possible environmental factors that influence the occurrence of ICP in genetically susceptible individuals. It has been postulated that the long-chain monounsaturated fatty acid erucic acid and/or selenium may play a role. Furthermore, the occurrence of ICP was exceptionally high in Chile a number of decades ago, but has drastically decreased more recently, suggesting that a change in diet or unknown lifestyle factor might play a role in ICP (2, 3).

The pathogenesis of cholestasis-associated pruritus is still poorly understood, however, based on research of the past 40 years and therapies that give relief of pruritus in a subset of patients, there is common understanding that:

- Potential pruritogens are located in the systemic circulation, as indicated by (partial) relief of pruritus after treatment with plasmapheresis, albumin dialysis or plasma separation/anion absorption.
- Direct or indirect pruritogens are excreted into bile and undergo enterohepatic circulation, as suggested by attenuation of pruritus in some patients after oral administration of anion exchange resins, and by inhibitors of the apical sodium-dependent bile acid transporter (ASBT) or effectively, but only transiently by nasobiliary drainage.
- Pruritogens are thought to be formed or biotransformed in the liver and/or the gut as indicated by effective treatment with the potent pregnane X receptor (PXR) agonist, rifampicin.
- Pruritogens affect the endogenous opioidergic and serotoninergic system, as suggested by antipruritic activity of opioid antagonists such as naltrexone and serotonin reuptake inhibitors such as sertraline.

In this review, we will discuss the current literature on cholestasis-associated pruritus, with a specific disquisition of the possible pruritogens. We will briefly deliberate the receptors involved in pruritus and current treatment options.

### Itch Signaling

Signaling of itch is often measured after provocation with histamine, chloroquine or cowhage, which activate the histamine 1 (H1R) or 4 (H4R) receptor (4), the Mas-related G-protein X1 in humans (MRGPRX1) or a3 in mice (mrgpra3) (5), and the protease-activated receptor 2 (PAR2) or 4 (PAR4) (6), respectively.

Histamine-induced itch signaling occurs through mechano-insensitive C-fibers (CMi) with unmyelinated nerve endings in the skin (7, 8). However, the majority of chronic itch types including cholestasis-associated pruritus cannot be relieved by anti-histamine treatment, and are therefore classified as non-histaminergic itch (9–13). Non-histaminergic itch in humans possibly signals through mechano-heat-sensitive C-fibers (CMH), as measured with cowhage provocation (14, 15).

Itch signals run from primary itch neurons in the skin through the dorsal root ganglia (DRG) to a secondary neuron in the dorsal horn of the spinal cord (16) with natriuretic peptide B (Nppb) and glutamate as the neurotransmitters (16–19). In the dorsal horn of the spinal cord, the secondary neuron crosses to the contralateral side and transmits the signal by gastrin-releasing peptide (GRP) to a tertiary neuron (16, 20, 21). This third neuron projects through the spinothalamic tract to the ventromedial nucleus of the thalamus.

In order to reduce itch, the central nervous system provokes an urge to create a local pain signal by scratching. This demonstrates that pain has an inhibitory effect on itch (22, 23). When pain is pharmacologically reduced, for example by morphine administration, itch frequently sets in at the region of reduced pain transmission (24, 25). This contra-mechanism is very likely fulfilled by inhibitory interneurons in the dorsal horn of the spinal cord, which create a constant tonus of inhibition by nociceptive to pruriceptive neurons. In genetic experiments with mice, it was shown that when these inhibitory interneurons are not formed, due to deletion of the gene for the transcription factor *Bhlhb5*, mice suffer from severe chronic itch (26).

Many receptors that are involved in pruritus are G-protein coupled receptors, which, after activation, cause an increase in intracellular calcium release and thereby activate PLC and PKC [reviewed by (27)]. PLC and PKC are often coupled to TRP-channels that amplify the intracellular cation wave and together with Na_V_ channels initiate an action potential, leading to itch sensation (28–31). Next to their role in coupled cation influx, these TRP-channels can also be activated directly by chemical, thermal and mechanical noxious stimuli (32–34).

### Pruritogens That Are Potentially Involved in Cholestatic Itch

#### Bile Salts

The most abundant components of bile are bile salts, which for a long time were and still are thought by some to act as pruritogens in cholestasis. There are several subspecies of bile salts with different properties. They can bind to the intracellular farnesoid X receptor (FXR) which is a nuclear receptor that regulates a considerable transcription network, and to the transmembrane G protein-coupled receptor TGR5 that upon activation induces cAMP synthesis. A specific subset of bile salts can even cause a response through the pregnane X receptor, vitamin D receptor and constitutive androstane receptor, thereby influencing bile salt synthesis and progression of cholestasis (35).

Bile salt levels are elevated in serum of cholestatic patients (36, 37). Elimination of bile with all its substances by nasobiliary drainage, or removal of albumin-bound substances (including bile salts) by albumin dialysis, often cause a tremendous diminution in the perception of itch, although this only lasts for a few weeks to months after the intervention (38–41). The results of these therapies clearly indicate that pruritogen(s) or progenitors of pruritogens are excreted in bile, but not necessarily that bile salts are causing cholestasis-associated pruritus.

Early research aimed at binding bile salts in the gut by using anion exchange resins (and, thereby, also bile salt sequestrants) like cholestyramine, which resulted in some reduction of pruritus (42–44). However, a more recent report indicates that the later developed, more potent anion exchange resin colesevelam does not improve pruritus more than placebo, despite the fact that bile salt levels were reduced by nearly 50% (45).

The group of Bunnett intensely studied itch signaling by bile salts. They proposed that itch signaling is mediated by the bile salt receptor TGR5. TGR5 expression was detected in the soma of DRG neurons of mice (but not in nerve fibers in the skin or the dorsal horn). Activation of DRG neurons led to the release of the neuropeptide gastrin-releasing peptide (GRP) and the opioid peptide leucine-enkephalin, which are thought to be itch and analgesia transmitters (46). A year later, the same group showed that the TGR5 receptor was co-expressed with the itch channel TRPA1 and activation of this axis induced scratch activity in mice (47). Indications for the involvement of TGR5 in itch signaling seem strong, but the role for bile salts as candidate pruritogens in relation to cholestasis-associated pruritus remains inconclusive. Several clinical studies have shown that there is no correlation between bile salts in serum, urine or skin and the intensity of pruritus (48–51). Pruritus is often one of the first symptoms of cholestatic liver disease, while bile salts levels are still relatively low (52, 53). During late stages of the disease, or in obstructive cholestasis, when bile salt levels can increase to high concentrations, pruritus is sometimes reported to subside (54).

Another receptor that is postulated to be involved in bile salt-induced itch signaling is the mas-related G protein receptor X4 (MRGPRX4). Mouse Mrgpr orthologs were not activated by bile salts and therefore the human receptor was expressed in mice and scratch activity was reported to be increased upon subcutaneous injection of the pruritogen chloroquine and the bile salt deoxycholate (DC) (55). However, injection with unconjugated bile salts is not very representative for cholestasis, since conjugated primary bile salts [like taurochenodeoxycholate (TCDC) and glycochenodeoxycholate (GCDC)] are increased during cholestasis whereas the pool size of conjugates of the secondary bile salt DC is reduced in cholestasis (56). Moreover, unconjugated bile salts diffuse into cells and cause increases in cytosolic free calcium (57, 58). In the same report, mice were treated with the hepatotoxicant α-naphthylisothiocyanate (ANIT), which is a known inducer of cholestasis, serious liver damage, including hepatocellular necrosis and liver fibrosis (59–62). Scratch activity was found to be increased in mice expressing MRGPRX4 compared to control animals, but only during the first 2 days of cholestasis (56), suggesting that this signaling undergoes fairly rapid desensitization. Such a short period of scratch behavior is not representative of the human situation, in which pruritus usually lasts for months and years. Moreover, in two other reports ANIT treatment did not lead to increased scratch activity [(63) and Langedijk et al., submitted].

A second, independent, report on MRGPRX4 showed that intradermal injection of 500 μg DC induced an acute itch sensation in healthy volunteers (64). These researchers showed that MRGPRX4 mRNA is expressed in 6–8% of human DRG neurons and co-expressed with H1R, TRPV1, and Na_v_1.7 voltage-gated sodium channel; which are all established itch-related channels. Since high concentrations of administered bile salts can lead to direct activation and degranulation of mast cells, and thereby release of histamine (65), the authors included the administration of antihistamines, which did not reduce DC-induced itch, indicating a non-histaminergic mechanism. However, DC, which showed to be the most potent ligand for MRGPRX4 among all tested bile salts, was not present in a different concentration in plasma of itchy vs. non-itchy patients with cholestasis (64). Other bile salts that showed a bigger difference were only weak agonists for MRGPRX4 (64).

Together, these results indicate that bile salts are involved in many processes, by binding to several receptors that are, among others, located on sensory neurons. However, as pruritogens for cholestatic itch, plasma bile salt concentrations do not correlate with clinical complaints of itch. The most prominent example against the theory of bile salts as pruritogens is represented by patients suffering from Na^+^-taurocholate cotransporting polypeptide (NTCP)-deficiency. The plasma bile salt levels of these patients can be extremely high, but patients do not complain of pruritus (66–69).

In conclusion, human clinical observations overrule all *in vivo* and *in vitro* research and pose the strongest argument against bile salts as dominant pruritogens in cholestatic itch.

#### Endogenous Opioids

The role of endogenous opioids in cholestasis-associated pruritus has recently been reviewed by Bergasa (70). Here, we want to discuss the most important arguments that support or dispute the role of endogenous opioids as causative pruritogens in cholestatic liver disease.

The central mechanism of pruritus is thought to include upregulation of the μ-opioid receptor system and suppression of the κ-opioid receptor system (71). One of the first observations in relation to opioids and pruritus is the fact that the μ-receptor agonist morphine can induce pruritus in humans (24, 72), while the μ-receptor antagonist naloxone can inhibit this reaction (73, 74). Endogenous opioid levels, including methionine enkephalin, leucine enkephalin and β-endorphin (μ-receptor agonists), were increased in plasma of bile duct ligated (BDL) animals and cholestatic patients with cirrhosis (75–77). However, in a more recent study the plasma levels of these μ-receptor agonists were comparable between cholestatic patients with and without pruritus, and were not increased in women with ICP compared to regular pregnancies (48). As was shown before in PBC patients, methionine-enkephalin concentrations correlate with the stage of disease but not with the severity of pruritus (75). Most studies into the beneficial effects of opioid antagonists involved few patients (in some cases even case reports) and/or were not placebo-controlled. Although treatment with μ-receptor antagonists like naloxone and naltrexone could reduce the level of pruritus in cholestatic patients, it can also lead to an opiate withdrawal-like reaction (73, 77–81).

Next to inhibiting the μ-opioid receptor, studies have been performed on activating the κ-opioid receptor system in mice, thereby reducing scratch activity induced by substance P, histamine or morphine (82, 83). The κ-receptor agonist nalfurafine has been studied in Japan, to treat pruritus in patients with chronic kidney disease (84, 85) and chronic liver disease (71, 86, 87). Treatment showed a partial decrease in levels of pruritus without any severe adverse events. The κ-opioid receptor agonist asimadoline is being tested in a phase II clinical trial to assess its efficacy on the relief of pruritus in patients with atopic dermatitis (88). For an overview on κ-opioid receptor agonists, see (89).

#### LPA/ATX

We have reported in earlier studies that lysophosphatidic acid (LPA) is a potential pruritogen in cholestasis (48, 90). LPA in serum of pregnant women with ICP and in serum of PBC patients with pruritus induced an increased Ca^2+^ response when applied to neuronal cells (48). Intradermal injection of LPA in the skin of mice initiated a scratch response, which confirmed a previous independent study (48, 91). The main production of LPA comes from the phospholipase D autotaxin (ATX), which catalyzes the hydrolysis of lysophosphatidylcholine (LPC) into LPA and choline (92–94). ATX (ENPP2) belongs to the family of ectonucleotide pyrophosphatases/phosphodiesterases (ENPP1-7) and promotes multiple functions like cell migration, angiogenesis and metastasis (95). A direct causal relation between plasma ATX and production of LPA is seen in ATX heterozygous mice, which show a reduction of 50% in ATX activity and plasma LPA levels, while ATX-deficient mice die prematurely due to vascular defects (96). High ATX mRNA expression in human tissues has been described for brain, ovary, lung and kidney, while enzyme activity has been detected in blood, cerebrospinal and seminal fluid, urine and saliva [reviewed in (97)]. So far, it is still unclear which of these organs contribute to the circulating ATX levels in human plasma and cerebrospinal fluid. Recently, our group has shown that human enteroendocrine cells of the small intestine are also a source of ATX (98).

LPA can activate neuronal cells, satellite glia cells and other cell types *via* six different LPA-specific receptors (LPAR1-6) (99–101). It has been described that LPA can induce neuropathic pain *via* LPAR1, LPAR3 and LPAR5 (102). However, in a study using the cheek injection mouse model Kittaka et al. (103) showed that LPA is a mediator of only itch rather than of pain. In this study, LPA was found to activate LPAR5 which, *via* intracellular phospholipase D activation, generates intracellular LPA that can activate TRPA1 and TRPV1, and thereby induce itch sensation.

The crystal structure of ATX shows an active site containing a hydrophilic groove, a hydrophobic lipid-binding pocket and a hydrophobic channel (tunnel) (104–106). This tunnel structure is able to selectively bind steroids and bile salts that lead to inhibition of ATX activity (107). The question remains whether more compounds involved in cholestasis can bind in this tunnel, and this way influence ATX activity.

Serum ATX levels are prominently increased in cholestatic patients with pruritus compared to cholestatic patients without pruritus, and in pregnant women with ICP compared to regular pregnancy. In our study, ATX activity correlated significantly with intensity of pruritus and with the effectiveness of treatment by rifampicin, MARS and nasobiliary drainage (48, 90). Serum ATX levels also indicate a therapeutic response to treatment with bezafibrate (108), prednisolone (109) and plasmapheresis (110). Even though ATX is not excreted into bile (48), interruption of the enterohepatic circulation by nasobiliary drainage and ASBT-inhibition still led to a decrease in both circulating ATX levels and pruritus scores (48, 111–113). These findings strongly indicate a tight relation between serum ATX levels and pruritus.

On the other hand, serum ATX levels are similarly increased in (pathological) conditions without itch, including regular pregnancy, some cancer entities and chronic viral hepatitis B and C. In several liver diseases accompanied by liver fibrosis (but not itch), serum ATX levels can also act as a marker of severity of liver injury [including non-alcoholic steatosis hepatitis (NASH) (114), PBC and PSC (115, 116), hepatitis B (117) and hepatitis C (118)]. A recent study reported that ATX levels correlate with fibrosis markers but not with frequency and severity of pruritus in PBC patients (119). A paradox seems to remain in the relation of serum ATX levels to pruritus. A common factor might be involved in both pruritus and liver fibrosis and therefore share ATX as a biomarker. Together, these results suggest that research into other cholestatic factors, that play a more dominant role in the initiation and/or potentiation of pruritus in cholestasis, is warranted.

#### Serotonin

Serotonin (5-HT) is a neurotransmitter in the central nervous system that can directly activate sensory neurons (120–122). Serotonin can be released from mast cells in the skin and can act as a pruritogen (123–125). The serotonergic system might be deregulated in cholestatic patients (126) and in patients with atopic dermatitis (127), leading to chronic pruritus. In rats with cholestasis induced by BDL, serotonin levels in the skin and spinal cord were significantly increased compared to sham mice, and enhanced scratching behavior was noted after mechanical and heat stimulation (128). In the absence of mechanical and heat stimuli, however, these mice showed no increased spontaneous scratching activity while they were clearly cholestatic.

Seven classes of 5-HT receptors have been identified (5-HT1-7) that comprise at least fifteen subtypes, of which all but one are metabotropic GPCRs (129, 130). 5-HT2 receptors are suggested to be important for itch perception (131, 132), by signaling through G_q/11_, which activates PLC (124, 129). Many pruritogens bind to metabotropic receptors on primary sensory neurons and are coupled to ionotropic channels *via* intracellular signaling pathways to allow sufficient current influx to generate action potentials (133). The cation channels TRPV1 and TRPA1 are often involved in itch transmission (134). A similar mechanism of a metabotropic receptor coupling to an ionotropic channel was found by Morita et al., where serotonin induces itch *via* activation of 5-HT7, which was shown to be coupled to TRPA1 (135). In a mouse model of atopic dermatitis, where mice were lacking 5-HT7 or TRPA1, the animals displayed reduced scratching. The authors suggest a role for 5-HT7 antagonists in the treatment of a variety of pathological itch conditions (135). By investigating the peripheral neuronal mechanisms underlying pruritus, Imamachi et al. showed that serotonin-induced pruritus in mice required the presence of PLCβ3 and TRPV1-expressing neurons, although not the TRPV1-channel itself (30). The TRPV4-channel, however, does seem to be involved as shown by reduced serotonin-evoked scratch bouts in TRPV4 knockout mice and wild type mice treated with a TRPV4 antagonist (131).

Selective serotonin reuptake inhibitors (SSRIs) have shown some effect against pruritus in cholestatic patients (136–139). They inhibit pre-synaptic reuptake of serotonin and might dull transmission of nociceptive stimuli through unmyelinated C-fibers (120, 140). When standard therapies have no effect on the reduction of pruritus, the SSRI sertraline is advised as a fourth line treatment (141). Notably, chronic pruritus is often associated with psychopathology, including anxiety and depression, where SSRIs may improve pruritus by treating underlying psychiatric comorbidities (142).

#### Histamine

Histamine is the best established pruritogen during acute allergic reactions, by activating histamine receptors (which are GPCRs) and TRP channels on sensory nerve endings. Antagonists of H1R are useful for the treatment of acute allergic pruritus, but are ineffective in the treatment of cholestasis-associated pruritus (141). Although plasma histamine levels have been reported to be slightly increased in cholestatic disease (143), in the study of Kremer et al., no correlation was found between plasma histamine levels and itch intensity in pruritic patients with ICP, PBC and other cholestatic disorders (48). Also skin changes consistent with histamine-mediated effects, like axon-reflex erythema (144), are not seen in cholestatic disease. Sedation with antihistamines may have a non-specific beneficial effect by improving sleep at night, but they reduce concentration during the day (145). Primary afferent neurons, responsible for histamine-induced itch in humans, belong to the group of mechano-insensitive unmyelinated C-fibers (7, 23, 146). However, a second group of C-fibers exist, that are insensitive to histamine, but sensitive to low intensity, high frequency electrical stimulation, which could explain itch without accompanying axon-reflex erythema (23, 144).

#### Bilirubin

Bilirubin is an essential component in bile and is the direct cause of jaundice in cholestatic diseases. It was recently hypothesized that bilirubin could act as a pruritogen by stimulating human MRGPRX4 and mouse mrgpra1 (147). MRGPRs are a family of GPCRs expressed in DRGs on primary sensory neurons (148, 149). In the study of Meixiong, the mouse receptor mrgpra1 and the human receptor MRGPRX4 were expressed in mouse DRGs that lacked expression of any other MRGPR (Mrgpr cluster KO mice). Bilirubin could activate 14 and 32% of the respective DRGs. Induction of cholestasis in mice by treatment with the hepatotoxicant ANIT led to hyperbilirubinemia and increased scratch activity, which was reduced in mrgpra1 KO and biliverdin reductase KO mice. From these results, the authors suggest that high serum bilirubin levels are involved in cholestasis-associated pruritus by binding to MRGPRX4 on sensory neurons (147).

Results from clinical observations, however, suggest otherwise. It has been shown multiple times that plasma bilirubin correlates poorly with pruritus intensity in cholestatic patients (50, 150). Pruritus is often observed while at the same time serum bilirubin levels are hardly elevated (141). Furthermore, many patients that suffer from pruritus are not jaundiced and vice versa, many jaundiced patients do not complain of itch. Patients with Dubin Johnson syndrome or Crigler Najjar syndrome type I have medium to very high plasma levels of bilirubin (conjugated and unconjugated, respectively) and hardly ever complain about itching (151). These results suggest that bilirubin is not a dominant pruritogen in cholestasis-associated pruritus.

#### Steroid Metabolites

Steroids like progesterone and estrogen might be involved in the pathogenesis of cholestasis-associated pruritus, particularly in the case of ICP. Progesterone and estrogen metabolites can be converted into steroids with a cholestatic potential (152). In women with ICP, serum concentrations of progesterone metabolites (pregnanediol sulfates) are elevated at 35–41 weeks of gestation (153, 154). These progesterone metabolites have been implicated as potential pruritogens from analyses of urine samples from ICP cases (155, 156). Progesterone metabolites can interact with the bile salt receptor FXR (153, 156), the bile salt transporter NTCP (157) and the bile salt export pump (BSEP) (158), thereby affecting bile salt homeostasis pathways and inhibiting hepatic bile salt uptake and efflux. This could possibly result in or contribute to cholestasis and hypercholanemia.

Three pregnanediol sulfates are found at increased concentrations in plasma during gestational weeks 9–15, prior to symptom onset of ICP (156). These concentrations were (weakly) associated with itch severity and were able to differentiate between women with ICP compared to those with benign pruritus gravidarum (156). One of these progesterone sulfates, 5β-pregnan-3α,-20α-diol-3-sulfate (PM3S), evoked a Tgr5-dependent scratch response in mice (156). Together, these results suggest that steroid metabolites might influence pruriception in cholestasis. Further research into steroid metabolites is needed to evaluate their mechanism in cholestasis-associated pruritus.

#### PAR2-Agonists

Protease-activated receptors (PARs) are implicated in somatosensory functions like itch and pain (159). They have been studied in relation to chronic itch in atopic dermatitis patients. PARs consist of four members, where PAR2 is highly expressed in keratinocytes and is thought to be involved in pruritus caused by atopic dermatitis (159–162). Activation of PAR2 in keratinocytes induces the release of pruritogenic cytokines that can induce itch through channels on sensory neurons (159). A recent study of Zhao et al. suggested that PAR2 mediates itch *via* TRPV3 signaling. Both PAR2 and TRPV1 were shown to be upregulated in the skin of patients with atopic dermatitis and in mouse models for atopic dermatitis (163).

PAR2 can be activated by the agonist SLIGRL and the proteases trypsin and tryptase. Skin application of those compounds have been shown to induce itch and scratch behavior in humans and in mice, respectively (162, 164–167). However, the shorter peptide, SLIGR, which also activates PAR2, did not induce scratching behavior but rather induced thermal hyperalgesia (168). Tryptase is a mast cell derived protease, which has been evaluated in cholestasis-associated pruritus, but a correlation with pruritus intensity could not be found (48). However, other proteases might be involved in cholestasis-associated pruritus that could signal through PAR2 and illicit itch sensation.

#### Gut Microbiome

Since cholestasis-associated pruritus is significantly improved by treatment with the antibiotic rifampicin (169), it seems plausible that gut microbiota are involved in cholestatic liver disease. The first link between gut microbiota and liver pathogenesis was made by Pereira et al., who hypothesized that neutrophil activators involved in inflammatory bowel disease, pass from the inflamed colon to the liver *via* the enterohepatic circulation (170). Biliary lactoferrin concentrations were increased in active ulcerative colitis and Crohn's disease, and fell with colectomy and disease remission. Patients with PSC are often also identified with inflammatory bowel disease. This suggests a potential role for gut-derived factors.

In a review of Li et al., several studies are discussed that suggest the important role of intestinal microbiota in the etiopathogenesis of cholestatic liver diseases by regulating metabolism and immune responses (171). A study in 2016 showed a reduction of several potential beneficial microbiota and enrichment of opportunistic pathogens in early-stage PBC patients compared to healthy controls (171, 172). Species richness was reduced in PBC patients, resulting in a shift in overall microbial diversity (171, 173). These results were ameliorated after ursodeoxycholic acid (UDCA) treatment. However, in both of these studies, cholestasis-associated pruritus was not measured. Probiotic studies have shown antipruritic effects in patients with atopic dermatitis (174, 175).

Recently, Hegade et al. performed a study in which they compared PBC-patients suffering from pruritus with non-symptomatic PBC patients and healthy volunteers (113). The gut microbiota showed no significant difference between those groups, suggesting that cholestasis-associated pruritus is not associated with a specific gut bacterial composition. However, after treatment with the ASBT-inhibitor linerixibat (GSK2330672), which reduces pruritus scores in PBC patients, fecal bacterial composition significantly changed from baseline. These changes might be due to the increased bile salt load in the colon resulting from ASBT inhibition (112, 113). Together, these studies do not support a major role for gut microbiota in the pathology of cholestasis-associated pruritus.

### Treatment of Cholestasis-Associated Pruritus

#### Evidence-Based Treatments

Evidence-based and experimental treatments for cholestasis-associated pruritus have recently been reviewed (176). Here, we will provide a brief overview. In general, treatment of pruritus can be associated with a considerable placebo effect when using subjective patient-reported outcomes (177, 178). In line with European Association for the Study of the Liver (141), patients with PBC, but also various other cholestatic liver diseases are treated with ursodeoxycholic acid (UDCA). UDCA exerts potent anticholestatic effects and improves biochemical surrogate markers of prognosis such as serum bilirubin or alkaline phosphatase (ALP), histological features (179–181) and liver transplantation-free survival in PBC and, according to most recently reported preliminary data, also in PSC. A beneficial effect of UDCA on pruritus, however, was not reported in large trials.

The non-specific peroxisome proliferator-activated receptor (PPAR) agonist bezafibrate has most recently been shown to effectively improve severe to moderate pruritus in 74 patients with PSC and PBC in a prospective, randomized, double-blind, placebo-controlled trial (182, 183). Bezafibrate has first been introduced as 2nd line treatment of PBC in Japan (184) in combination with UDCA in patients incompletely responding to UDCA alone. Its anticholestatic effect and that of the PPARα agonist fenofibrate has since then been documented in various small trials (185), and finally in a prospective, randomized, placebo-controlled study in 100 patients with PBC treated over 2 years (108, 186) convincingly confirmed. In this trial, a minor antipruritic effect of bezafibrate represented one of the favorable secondary observations in PBC patients with mild pruritus. A case series describing an antipruritic effect of bezafibrate was published in 2018 (187). It remains unclear at present whether bezafibrate is superior to the selective PPARα agonist fenofibrate, which also exerts anticholestatic and antipruritic effects in PBC. With its antipruritic, anticholestatic, and anti-inflammatory effects in fibrosing cholangiopathies such as PSC or PBC and its safety profile, bezafibrate—and potentially other PPAR agonists—should become the 1st line treatment in cholestasis-associated pruritus in the future.

The antibiotic rifampicin is a potent pregnane X receptor (PXR) agonist, and, thereby, inducer of phase 1 and phase 2 biotransformation enzymes including cytochrome P450 3A4, a major drug-metabolizing enzyme. Rifampicin is widely used to reduce cholestasis-associated pruritus and is recommended as a second line antipruritic agent (81, 188). It induces biotransformation of many endogenous and exogenous compounds by phase 1 (e.g., hydroxylation) and phase 2 (e.g., glucuronidation) reactions (189–191) to more water-soluble molecules which can then be excreted *via* the renal route. The antibacterial effects of rifampicin on intestinal flora may alter metabolization and absorption of primary and secondary bile salts and pruritogens (188). Additionally, rifampicin can downregulate ATX expression *via* PXR-dependent mechanisms and, thereby, reduce formation of the pruritogen LPA (90). Currently, it is still unclear whether one or the other of these effects plays a prominent role in the anti-pruritic action of rifampicin. Adverse effects of rifampicin include discoloring of body fluids and teeth and resistance against rifampicin-sensitive bacteria. Use of rifampicin for over more than 2 weeks may enhance the risk of hepatotoxicity and therefore requires strict guidance in patients with liver disease (141).

As third-line treatment for pruritus, oral opioid antagonists like naltrexone and naloxone are used (81, 192, 193). Endogenous opioids were found to be elevated in plasma of patients with acute liver disease and cirrhosis with ascites (77) but Kremer et al. did not observe a correlation between itch and plasma opioid activity (48). Opioid antagonists are able to reduce pruritus (77, 193); however, they also can cause severe side effects similar to opioid withdrawal symptoms (79) and should be started at low daily doses.

Patients that are resistant to above-mentioned treatments can receive the selective serotonin reuptake inhibitor (SSRI) sertraline as a fourth-line treatment (137). This antidepressant can alter potential pruritic pathways involving serotonin.

In the past, cholestyramine was used as the first-line recommended treatment against pruritus. Cholestyramine is an anion-exchange resin that can bind and sequester numerous amphiphilic compounds including bile salts and (other?) potential pruritogens in the intestine (43, 194, 195). Despite the long history of use, evidence for efficacy of this treatment modality is moderate at best (141, 196). In addition, cholestyramine quite often causes gastrointestinal complaints. In a prospective, randomized, placebo-controlled trial, a more potent anion-exchange resin, colesevelam, was able to reduce bile salt levels by nearly 50%, but no effect was observed on intensity of pruritus when compared to placebo (45). Therefore, we conclude that anion-exchange resins should not anymore be ranked among evidence-based, recommended treatment options and rather should have a role as escape medication when other (above discussed) antipruritic medications fail or are not tolerated.

#### Invasive Treatments

Invasive treatments are indicated for patients that do not respond to abovementioned medical drug therapies. Patients can undergo extracorporeal albumin dialysis (41, 182), plasmapheresis (110, 183–185) and biliary drainage (38–40). These treatments, if effective, only last for days or a few weeks and in exceptional cases months, after which pruritus returns. Surgical partial internal/external biliary diversion can be a permanent solution in children when performed before advanced liver disease has developed (186). Liver transplantation can be performed as a last resort for severe cholestasis-associated pruritus refractory to medical and invasive therapeutic approaches.

#### Experimental Treatments

Pruritogens might undergo enterohepatic circulation in a similar way as bile salts do. The reuptake of bile salts and other compounds in the ileum, through the intestinal apical sodium-dependent bile acid transporter (ASBT), can be reduced by the use of ASBT-inhibitors and can give a strong reduction of itch sensation in patients. Several inhibitory pharmaceuticals have been developed, which have minimal systemic absorption, thereby only affecting absorption in the intestine. More recent ASBT inhibitors studied include linerixibat (GSK2330672) (112, 113), maralixibat (SHP625) (178) and odevixibat (A4250) (111). Clinical trials with cholestatic patients suffering from pruritus showed promising, but equivocal, results in reducing pruritus (111–113, 178). Frequent adverse events were diarrhea and abdominal discomfort possibly due to increased bile salt load in the large intestine. Notably, the majority of these adverse events resolved over time in one of the studies (178).

In a placebo-controlled trial the effect of the κ-opioid antagonist nalfurafine was found to mildly reduce cholestasis-associated itch in 318 patients with chronic cholestasis-associated itch without major side effects (86).

Since serum ATX levels correlate well with itch intensity in cholestatic patients, and the ATX product LPA can act as a pruritogen (48, 90), future therapy for cholestasis-associated pruritus might include ATX-inhibitors [reviewed in (187)]. Current research on ATX and ATX-inhibition is predominantly performed in the cancer and inflammation fields (197, 198). The ATX-LPA axis is involved in multiple functions, including cell migration, angiogenesis and metastasis (95). Future studies will have to study the safety and efficacy of ATX-inhibitors and their potential role in cholestasis-associated pruritus.

## Challenges and Future Perspectives of Cholestasis-Associated Pruritus Research

Patients with cholestatic diseases often suffer from severe, chronic pruritus, which impairs their quality of life. It is up to researchers to unravel the mechanism of cholestasis-associated pruritus, and even more importantly, to provide a treatment to reduce or resolve this burden.

This review highlights the fact that for cholestasis-associated pruritus there are many possible pruritogens, of which several are likely to play a role simultaneously. Due to different theories on the cause of cholestasis-associated pruritus, also different treatments exist of which none has yet shown to represent the final solution. Research in this field will have to continue in order to clarify the mechanism and to provide a satisfying treatment for patients with cholestasis-associated pruritus.

One of the biggest challenges in cholestasis-associated pruritus research is the possible multi-organ aspect. Besides the affected liver, multiple other organs play a potential role in cholestasis-associated pruritus, including the intestine with its enterohepatic circulation of multiple unknown compounds, the blood with all its proteins, hormones and lipids, the skin with its receptors, barriers and nerves, and the brain of which still a lot is unknown. All of these aspects can affect the production, transport, accumulation, binding, metabolism, and excretion of potential pruritogen(s).

The next questions arise; are pruritogens produced in a higher amount in patients with cholestatic disease as compared to healthy people as a consequence of cholestasis, or are they normally present and do they accumulate due to cholestatic circumstances? Is it possible that there is increased production of pruritogens as a secondary effect of accumulation of non-pruritogens? Bile formation is an important way of eliminating waste products; however, many compounds in bile are recirculated in the enterohepatic circulation, which opens the possibility of accumulation during cholestasis. We can interrupt the complete enterohepatic circulation [by anion-exchange resins, nasobiliary drainage, ASBT-inhibition or partial external biliary diversion (PEBD)], or remove hypothetical compounds from the circulation (by plasmapheresis or albumin dialysis), but we still do not know for which compounds elimination is essential. Based on the most recent studies, we would expect higher levels of pruritogens in plasma (and possibly even in bile) of patients with chronic pruritus compared to people without chronic pruritus, since elimination or filtration provides relief of pruritus. However, the possibility remains that pruritus is not caused by a change in the level of pruritogens but rather the sensitivity to them. Thus, in patients with cholestasis-associated pruritus there might be a change in sensory signaling involving receptor expression in neurons or the skin. These alternatives need to be investigated in detail still.

Another challenge in pruritus research is the way of measuring pruritus (in mice and men), which is best analyzed in a mechanic or sensory way, but mental experience is also a potent factor in human patients with pruritus. The amount or intensity of pruritus needs to be expressed in an objective value which is comparable between individuals, and not affected by side effects like emotions, mental stress or fatigue. An important challenge in pruritus research is correcting for the placebo effect of possible treatment. Several studies have shown that treatment with placebo already improves itch sensation by a great deal. Hence, a placebo group should always be included.

Detection of pruritogens involved in cholestasis might find its future in untargeted metabolomic research. All metabolites of bile, serum and possibly skin can be measured and compared between groups. The question remains whether all metabolites can yet be identified and traced back to their origin, but future research will expand the pool of identified metabolites. This approach might reveal a more specific target to unravel the mechanisms and develop a better treatment for cholestasis-associated pruritus.

## Author Contributions

JL wrote the review with intellectual guidance by UB and RO. All authors contributed to the article and approved the submitted version.

## Conflict of Interest

The authors declare that the research was conducted in the absence of any commercial or financial relationships that could be construed as a potential conflict of interest.

## Abbreviations

5-HT: serotonin
γGT: γ-glutamyltransferase
ANIT: α-naphthylisothiocyanate
AP: alkaline phosphatase
ASBT: apical sodium-dependent bile salt transporter
ATX: autotaxin
BSEP: bile salt export pump
CMH: mechano-heat-sensitive C-fiber
CMi: mechano-insensitive C-fiber
DC: deoxycholate
DRG: dorsal root ganglion
EASL: European Association for the Study of the Liver
ENPP: ectonucleotide pyrophosphatase
FXR: farnesoid X receptor
GCDC: glycochenodeoxycholate
GPCR: G-protein coupled receptor
GRP: gastrin-releasing peptide
H1R: histamine 1 receptor
H4R: histamine 4 receptor
ICP: intrahepatic cholestasis of pregnancy
IPF: idiopathic pulmonary fibrosis
LPA: lysophosphatidic acid
LPC: lysophosphatidylcholine
Mrgpra3: mas-related G-protein a3
MRGPRX1: mas-related G-protein X1
MRGPRX4: mas-related G-protein X4
NASH: non-alcoholic steatosis hepatitis
Nppb: natriuretic peptide B
NTCP: Na^+^-taurocholate cotransporting polypeptide
PPAR: peroxisome proliferator-activated receptor
PAR2: protease-activated receptor 2
PAR4: protease-activated receptor 4
PBC: primary biliary cholangitis
PEBD: partial external biliary diversion
PKC: protein kinase C
PLC: phospholipase C
PM3S: progesterone metabolite 3 sulfate
PSC: primary sclerosing cholangitis
PFIC: progressive familial intrahepatic cholestasis
PXR: pregnane X receptor
SSRI: selective serotonin reuptake inhibitor
TCDC: taurochenodeoxycholate
TGR5: G-protein coupled bile salt receptor
TRPA1: transient receptor potential ankyrin 1
TRPV1: transient receptor potential vanilloid 1
UDCA: ursodeoxycholic acid.

## References

[1] Beuers U Kremer AE Bolier R Elferink RP. Pruritus in cholestasis: facts and fiction. Hepatology. (2014) 60:399–407. 10.1002/hep.26909 24807046

[2] Arrese M Macias RI Briz O Perez MJ Marin JJ. Molecular pathogenesis of intrahepatic cholestasis of pregnancy. Expert Rev Mol Med. (2008) 10:e9. 10.1017/S1462399408000628 18371245

[3] Reyes H Sulfated progesterone metabolites in the pathogenesis of intrahepatic cholestasis of pregnancy: another loop in the ascending spiral of medical knowledge. Hepatology. (2016) 63:1080–2. 10.1002/hep.28365 26599133

[4] Bell JK McQueen DS Rees JL. Involvement of histamine H4 and H1 receptors in scratching induced by histamine receptor agonists in Balb C mice. Br J Pharmacol. (2004) 142:374–80. 10.1038/sj.bjp.0705754 15066908 PMC1574944

[5] Liu Q Tang Z Surdenikova L Kim S Patel KN Kim A. Sensory neuron-specific GPCR Mrgprs are itch receptors mediating chloroquine-induced pruritus. Cell. (2009) 139:1353–65. 10.1016/j.cell.2009.11.034 20004959 PMC2989405

[6] Reddy VB Iuga AO Shimada SG LaMotte RH Lerner EA. Cowhage-evoked itch is mediated by a novel cysteine protease: a ligand of protease-activated receptors. J Neurosci. (2008) 28:4331–5. 10.1523/JNEUROSCI.0716-08.2008 18434511 PMC2659338

[7] Schmelz M Schmidt R Bickel A Handwerker HO Torebjork HE. Specific C-receptors for itch in human skin. J Neurosci. (1997) 17:8003–8. 10.1523/JNEUROSCI.17-20-08003.1997 9315918 PMC6793906

[8] Andrew D Craig AD. Spinothalamic lamina I neurons selectively sensitive to histamine: a central neural pathway for itch. Nat Neurosci. (2001) 4:72–7. 10.1038/82924 11135647

[9] Yosipovitch G Rosen JD Hashimoto T. Itch: From mechanism to (novel) therapeutic approaches. J Allergy Clin Immunol. (2018) 142:1375–90. 10.1016/j.jaci.2018.09.005 30409247

[10] Andersen HH Elberling J Solvsten H Yosipovitch G Arendt-Nielsen L. Nonhistaminergic and mechanical itch sensitization in atopic dermatitis. Pain. (2017) 158:1780–91. 10.1097/j.pain.0000000000000980 28614190

[11] Davidson S Zhang X Yoon CH Khasabov SG Simone DA Giesler GJ Jr. The itch-producing agents histamine and cowhage activate separate populations of primate spinothalamic tract neurons. J Neurosci. (2007) 27:10007–14. 10.1523/JNEUROSCI.2862-07.2007 17855615 PMC3008349

[12] Shelley WB Arthur RP. Mucunain, the active pruritogenic proteinase of cowhage. Science. (1955) 122:469–70. 10.1126/science.122.3167.469 13255877

[13] Shelley WB Arthur RP. Studies on cowhage (*Mucuna pruriens*) and its pruritogenic proteinase, mucunain. AMA Arch Derm. (1955) 72:399–406. 10.1001/archderm.1955.03730350001001 13257913

[14] Johanek LM Meyer RA Friedman RM Greenquist KW Shim B Borzan J. A role for polymodal C-fiber afferents in nonhistaminergic itch. J Neurosci. (2008) 28:7659–69. 10.1523/JNEUROSCI.1760-08.2008 18650342 PMC2564794

[15] Namer B Carr R Johanek LM Schmelz M Handwerker HO Ringkamp M. Separate peripheral pathways for pruritus in man. J Neurophysiol. (2008) 100:2062–9. 10.1152/jn.90482.2008 18562548 PMC2576220

[16] Mishra SK Hoon MA. The cells and circuitry for itch responses in mice. Science. (2013) 340:968–71. 10.1126/science.1233765 23704570 PMC3670709

[17] Koga K Chen T Li XY Descalzi G Ling J Gu J. Glutamate acts as a neurotransmitter for gastrin releasing peptide-sensitive and insensitive itch-related synaptic transmission in mammalian spinal cord. Mol Pain. (2011) 7:47. 10.1186/1744-8069-7-47 21699733 PMC3132725

[18] Lagerstrom MC Rogoz K Abrahamsen B Persson E Reinius B Nordenankar K. VGLUT2-dependent sensory neurons in the TRPV1 population regulate pain and itch. Neuron. (2010) 68:529–42. 10.1016/j.neuron.2010.09.016 21040852 PMC3052264

[19] Liu Y Abdel Samad O Zhang L Duan B Tong Q Lopes C. VGLUT2-dependent glutamate release from nociceptors is required to sense pain and suppress itch. Neuron. (2010) 68:543–56. 10.1016/j.neuron.2010.09.008 21040853 PMC2991105

[20] Sun YG Chen ZF. A gastrin-releasing peptide receptor mediates the itch sensation in the spinal cord. Nature. (2007) 448:700–3. 10.1038/nature06029 17653196

[21] Sun YG Zhao ZQ Meng XL Yin J Liu XY Chen ZF. Cellular basis of itch sensation. Science. (2009) 325:1531–4. 10.1126/science.1174868 19661382 PMC2786498

[22] Yosipovitch G Bernhard JD. Clinical practice. Chronic pruritus. N Engl J Med. (2013) 368:1625–34. 10.1056/NEJMcp1208814 23614588

[23] Ikoma A Steinhoff M Stander S Yosipovitch G Schmelz M. The neurobiology of itch. Nat Rev Neurosci. (2006) 7:535–47. 10.1038/nrn1950 16791143

[24] Ballantyne JC Loach AB Carr DB. Itching after epidural and spinal opiates. Pain. (1988) 33:149–60. 10.1016/0304-3959(88)90085-1 2837714

[25] Atanassoff PG Brull SJ Zhang J Greenquist K Silverman DG Lamotte RH. Enhancement of experimental pruritus and mechanically evoked dysesthesiae with local anesthesia. Somatosens Mot Res. (1999) 16:291–8. 10.1080/08990229970357 10632026

[26] Ross SE Mardinly AR McCord AE Zurawski J Cohen S Jung C. Loss of inhibitory interneurons in the dorsal spinal cord and elevated itch in Bhlhb5 mutant mice. Neuron. (2010) 65:886–98. 10.1016/j.neuron.2010.02.025 20346763 PMC2856621

[27] Dong X Dong X. Peripheral and central mechanisms of itch. Neuron. (2018) 98:482–94. 10.1016/j.neuron.2018.03.023 29723501 PMC6022762

[28] Wilson SR Gerhold KA Bifolck-Fisher A Liu Q Patel KN Dong X. TRPA1 is required for histamine-independent, Mas-related G protein-coupled receptor-mediated itch. Nat Neurosci. (2011) 14:595–602. 10.1038/nn.2789 21460831 PMC3181150

[29] Amadesi S Cottrell GS Divino L Chapman K Grady EF Bautista F. Protease-activated receptor 2 sensitizes TRPV1 by protein kinase Cepsilon- and A-dependent mechanisms in rats and mice. J Physiol. (2006) 575:555–71. 10.1113/jphysiol.2006.111534 16793902 PMC1819458

[30] Imamachi N Park GH Lee H Anderson DJ Simon MI Basbaum AI. TRPV1-expressing primary afferents generate behavioral responses to pruritogens via multiple mechanisms. Proc Natl Acad Sci USA. (2009) 106:11330–5. 10.1073/pnas.0905605106 19564617 PMC2708751

[31] Jurcakova D Ru F Kollarik M Sun H Krajewski J Undem BJ. Voltage-gated sodium channels regulating action potential generation in itch-, nociceptive-, and low-threshold mechanosensitive cutaneous C-fibers. Mol Pharmacol. (2018) 94:1047–56. 10.1124/mol.118.112839 29941667

[32] Kittaka H Tominaga M. The molecular and cellular mechanisms of itch and the involvement of TRP channels in the peripheral sensory nervous system and skin. Allergol Int. (2017) 66:22–30. 10.1016/j.alit.2016.10.003 28012781

[33] Moore C Gupta R Jordt SE Chen Y Liedtke WB. Regulation of pain and itch by TRP channels. Neurosci Bull. (2018) 34:120–42. 10.1007/s12264-017-0200-8 29282613 PMC5799130

[34] Xie Z Hu H. TRP channels as drug targets to relieve itch. Pharmaceuticals (Basel). (2018) 11:100. 10.3390/ph11040100 30301231 PMC6316386

[35] Schaap FG Trauner M Jansen PL. Bile acid receptors as targets for drug development. Nat Rev Gastroenterol Hepatol. (2014) 11:55–67. 10.1038/nrgastro.2013.151 23982684

[36] Trottier J Bialek A Caron P Straka RJ Heathcote J Milkiewicz P. Metabolomic profiling of 17 bile acids in serum from patients with primary biliary cirrhosis and primary sclerosing cholangitis: a pilot study. Dig Liver Dis. (2012) 44:303–10. 10.1016/j.dld.2011.10.025 22169272

[37] Woolbright BL Dorko K Antoine DJ Clarke JI Gholami P Li F. Bile acid-induced necrosis in primary human hepatocytes and in patients with obstructive cholestasis. Toxicol Appl Pharmacol. (2015) 283:168–77. 10.1016/j.taap.2015.01.015 25636263 PMC4361327

[38] Hegade VS Krawczyk M Kremer AE Kuczka J Gaouar F Kuiper EM. The safety and efficacy of nasobiliary drainage in the treatment of refractory cholestatic pruritus: a multicentre European study. Aliment Pharmacol Ther. (2016) 43:294–302. 10.1111/apt.13449 26526892

[39] Beuers U Gerken G Pusl T. Biliary drainage transiently relieves intractable pruritus in primary biliary cirrhosis. Hepatology. (2006) 44:280–1. 10.1002/hep.21271 16799978

[40] Stapelbroek JM van Erpecum KJ Klomp LW Venneman NG Schwartz TP van Berge Henegouwen GP. Nasobiliary drainage induces long-lasting remission in benign recurrent intrahepatic cholestasis. Hepatology. (2006) 43:51–3. 10.1002/hep.20998 16374853

[41] Pares A Herrera M Aviles J Sanz M Mas A. Treatment of resistant pruritus from cholestasis with albumin dialysis: combined analysis of patients from three centers. J Hepatol. (2010) 53:307–12. 10.1016/j.jhep.2010.02.031 20580987

[42] Carey JB Jr. Bile acids in the serum of jaundiced patients. Gastroenterology. (1961) 41:285–7. 10.1016/S0016-5085(19)35143-1 13690774

[43] Datta DV Sherlock S. Cholestyramine for long term relief of the pruritus complicating intrahepatic cholestasis. Gastroenterology. (1966) 50:323–32. 10.1016/S0016-5085(66)80071-9 5905351

[44] Oster ZH Rachmilewitz EA Moran E Stein Y. Relief of pruritus by cholestyramine in chronic liver disease. Isr J Med Sci. (1965) 1:599–606. 5842274

[45] Kuiper EM van Erpecum KJ Beuers U Hansen BE Thio HB de Man RA. The potent bile acid sequestrant colesevelam is not effective in cholestatic pruritus: results of a double-blind, randomized, placebo-controlled trial. Hepatology. (2010) 52:1334–40. 10.1002/hep.23821 20683930

[46] Alemi F Kwon E Poole DP Lieu T Lyo V Cattaruzza F. The TGR5 receptor mediates bile acid-induced itch and analgesia. J Clin Invest. (2013) 123:1513–30. 10.1172/JCI64551 23524965 PMC3613908

[47] Lieu T Jayaweera G Zhao P Poole DP Jensen D Grace M. The bile acid receptor TGR5 activates the TRPA1 channel to induce itch in mice. Gastroenterology. (2014) 147:1417–28. 10.1053/j.gastro.2014.08.042 25194674 PMC4821165

[48] Kremer AE Martens JJ Kulik W Rueff F Kuiper EM van Buuren HR. Lysophosphatidic acid is a potential mediator of cholestatic pruritus. Gastroenterology. (2010) 139:1008–18. 10.1053/j.gastro.2010.05.009 20546739

[49] Bartholomew TC Summerfield JA Billing BH Lawson AM Setchell KD. Bile acid profiles of human serum and skin interstitial fluid and their relationship to pruritus studied by gas chromatography-mass spectrometry. Clin Sci (Lond). (1982) 63:65–73. 10.1042/cs0630065 7083767

[50] Freedman MR Holzbach RT Ferguson DR. Pruritus in cholestasis—no direct causative role for bile-acid retention. Am J Med. (1981) 70:1011–6. 10.1016/0002-9343(81)90857-3 7234869

[51] Ghent CN Bloomer JR Klatskin G. Elevations in skin tissue levels of bile-acids in human cholestasis—relation to serum levels and to pruritus. Gastroenterology. (1977) 73:1125–30. 10.1016/S0016-5085(19)31870-0 908491

[52] Geenes V Williamson C. Intrahepatic cholestasis of pregnancy. World J Gastroenterol. (2009) 15:2049–66. 10.3748/wjg.15.2049 19418576 PMC2678574

[53] Kremer AE Beuers U Oude-Elferink RP Pusl T. Pathogenesis and treatment of pruritus in cholestasis. Drugs. (2008) 68:2163–82. 10.2165/00003495-200868150-00006 18840005

[54] Murphy GM Ross A Billing BH. Serum bile acids in primary biliary cirrhosis. Gut. (1972) 13:201–6. 10.1136/gut.13.3.201 5024725 PMC1412131

[55] Meixiong J Vasavda C Snyder SH Dong X. MRGPRX4 is a G protein-coupled receptor activated by bile acids that may contribute to cholestatic pruritus. Proc Natl Acad Sci USA. (2019) 116:10525–30. 10.1073/pnas.1903316116 31068464 PMC6535009

[56] Beuers U Spengler U Zwiebel FM Pauletzki J Fischer S Paumgartner G. Effect of ursodeoxycholic acid on the kinetics of the major hydrophobic bile acids in health and in chronic cholestatic liver disease. Hepatology. (1992) 15:603–8. 10.1002/hep.1840150409 1551637

[57] Dziki AJ Batzri S Harmon JW Molloy M. Cellular hypercalcemia is an early event in deoxycholate injury of rabbit gastric mucosal cells. Am J Physiol. (1995) 269:G287–96. 10.1152/ajpgi.1995.269.2.G287 7653570

[58] Lau BW Colella M Ruder WC Ranieri M Curci S Hofer AM. Deoxycholic acid activates protein kinase C and phospholipase C *vi*a increased Ca2+ entry at plasma membrane. Gastroenterology. (2005) 128:695–707. 10.1053/j.gastro.2004.12.046 15765405

[59] Joshi N Ray JL Kopec AK Luyendyk JP. Dose-dependent effects of alpha-naphthylisothiocyanate disconnect biliary fibrosis from hepatocellular necrosis. J Biochem Mol Toxicol. (2017) 31:1–7. 10.1002/jbt.21834 27605088 PMC5509966

[60] Golbar HM Izawa T Ichikawa C Tanaka M Juniantito V Sawamoto O. Slowly progressive cholangiofibrosis induced in rats by alpha-naphthylisothiocyanate (ANIT), with particular references to characteristics of macrophages and myofibroblasts. Exp Toxicol Pathol. (2013) 65:825–35. 10.1016/j.etp.2012.12.001 23298557

[61] Tjandra K Sharkey KA Swain MG. Progressive development of a Th1-type hepatic cytokine profile in rats with experimental cholangitis. Hepatology. (2000) 31:280–90. 10.1002/hep.510310204 10655247

[62] Xu J Lee G Wang H Vierling JM Maher JJ. Limited role for CXC chemokines in the pathogenesis of alpha-naphthylisothiocyanate-induced liver injury. Am J Physiol Gastrointest Liver Physiol. (2004) 287:G734–41. 10.1152/ajpgi.00300.2003 15130876 PMC3622103

[63] Cipriani S Renga B D'Amore C Simonetti M De Tursi AA Carino A. Impaired itching perception in murine models of cholestasis is supported by dysregulation of GPBAR1 signaling. PLoS ONE. (2015) 10:e0129866. 10.1371/journal.pone.0129866 26177448 PMC4503431

[64] Yu H Zhao T Liu S Wu Q Johnson O Wu Z. MRGPRX4 is a bile acid receptor for human cholestatic itch. Elife. (2019) 8:48431. 10.7554/eLife.48431 31500698 PMC6773440

[65] Quist RG Ton-Nu HT Lillienau J Hofmann AF Barrett KE. Activation of mast cells by bile acids. Gastroenterology. (1991) 101:446–56. 10.1016/0016-5085(91)90024-F 1712330

[66] Vaz FM Paulusma CC Huidekoper H de Ru M Lim C Koster J. Sodium taurocholate cotransporting polypeptide (SLC10A1) deficiency: conjugated hypercholanemia without a clear clinical phenotype. Hepatology. (2015) 61:260–7. 10.1002/hep.27240 24867799

[67] Van Herpe F Waterham HR Adams CJ Mannens M Bikker H Vaz FM. NTCP deficiency and persistently raised bile salts: an adult case. J Inherit Metab Dis. (2017) 40:313–5. 10.1007/s10545-017-0031-9 28283843

[68] Erlinger S NTCP deficiency: a new inherited disease of bile acid transport. Clin Res Hepatol Gastroenterol. (2015) 39:7–8. 10.1016/j.clinre.2014.07.011 25193235

[69] Vaz FM Huidekoper HH Paulusma CC. Extended abstract: deficiency of sodium taurocholate cotransporting polypeptide (SLC10A1): a new inborn error of metabolism with an attenuated phenotype. Dig Dis. (2017) 35:259–60. 10.1159/000450984 28249272

[70] Bergasa NV. The pruritus of cholestasis: from bile acids to opiate agonists: relevant after all these years. Med Hypotheses. (2018) 110:86–9. 10.1016/j.mehy.2017.11.002 29317077

[71] Kamimura K Yokoo T Kamimura H Sakamaki A Abe S Tsuchiya A. Long-term efficacy and safety of nalfurafine hydrochloride on pruritus in chronic liver disease patients: patient-reported outcome based analyses. PLoS ONE. (2017) 12:e0178991. 10.1371/journal.pone.0178991 28604788 PMC5467861

[72] Cousins MJ Mather LE. Intrathecal and epidural administration of opioids. Anesthesiology. (1984) 61:276–310. 10.1097/00000542-198409000-00008 6206753

[73] Bergasa NV Talbot TL Alling DW Schmitt JM Walker EC Baker BL. A controlled trial of naloxone infusions for the pruritus of chronic cholestasis. Gastroenterology. (1992) 102:544–9. 10.1016/0016-5085(92)90102-5 1732125

[74] Bernstein JE Swift RM Soltani K Lorincz AL. Antipruritic effect of an opiate antagonist, naloxone hydrochloride. J Invest Dermatol. (1982) 78:82–3. 10.1111/1523-1747.ep12497974 7054311

[75] Spivey JR Jorgensen RA Gores GJ Lindor KD. Methionine-enkephalin concentrations correlate with stage of disease but not pruritus in patients with primary biliary cirrhosis. Am J Gastroenterol. (1994) 89:2028–32. 7942730

[76] Swain MG Rothman RB Xu H Vergalla J Bergasa NV Jones EA. Endogenous opioids accumulate in plasma in a rat model of acute cholestasis. Gastroenterology. (1992) 103:630–5. 10.1016/0016-5085(92)90857-U 1634078

[77] Thornton JR Losowsky MS. Opioid peptides and primary biliary cirrhosis. BMJ. (1988) 297:1501–4. 10.1136/bmj.297.6662.1501 3147046 PMC1835218

[78] Jones EA Dekker LR. Florid opioid withdrawal-like reaction precipitated by naltrexone in a patient with chronic cholestasis. Gastroenterology. (2000) 118:431–2. 10.1016/S0016-5085(00)70225-3 10648471

[79] Mansour-Ghanaei F Taheri A Froutan H Ghofrani H Nasiri-Toosi M Bagherzadeh AH. Effect of oral naltrexone on pruritus in cholestatic patients. World J Gastroenterol. (2006) 12:1125–8. 10.3748/wjg.v12.i7.1125 16534857 PMC4087908

[80] Shawcross DL Jalan R. Delayed opioid withdrawal-like reaction in primary biliary cirrhosis following naloxone therapy. Gastroenterology. (2001) 121:743–4. 10.1053/gast.2001.27714 11547785

[81] Tandon P Rowe BH Vandermeer B Bain VG. The efficacy and safety of bile Acid binding agents, opioid antagonists, or rifampin in the treatment of cholestasis-associated pruritus. Am J Gastroenterol. (2007) 102:1528–36. 10.1111/j.1572-0241.2007.01200.x 17403073

[82] Togashi Y Umeuchi H Okano K Ando N Yoshizawa Y Honda T. Antipruritic activity of the kappa-opioid receptor agonist, TRK-820. Eur J Pharmacol. (2002) 435:259–64. 10.1016/S0014-2999(01)01588-6 11821035

[83] Umeuchi H Togashi Y Honda T Nakao K Okano K Tanaka T. Involvement of central mu-opioid system in the scratching behavior in mice, and the suppression of it by the activation of kappa-opioid system. Eur J Pharmacol. (2003) 477:29–35. 10.1016/j.ejphar.2003.08.007 14512095

[84] Kumagai H Ebata T Takamori K Muramatsu T Nakamoto H Suzuki H. Effect of a novel kappa-receptor agonist, nalfurafine hydrochloride, on severe itch in 337 haemodialysis patients: a Phase III, randomized, double-blind, placebo-controlled study. Nephrol Dial Transplant. (2010) 25:1251–7. 10.1093/ndt/gfp588 19926718

[85] Wikstrom B Gellert R Ladefoged SD Danda Y Akai M Ide K. Kappa-opioid system in uremic pruritus: multicenter, randomized, double-blind, placebo-controlled clinical studies. J Am Soc Nephrol. (2005) 16:3742–7. 10.1681/ASN.2005020152 16251241

[86] Kumada H Miyakawa H Muramatsu T Ando N Oh T Takamori K. Efficacy of nalfurafine hydrochloride in patients with chronic liver disease with refractory pruritus: a randomized, double-blind trial. Hepatol Res. (2017) 47:972–82. 10.1111/hepr.12830 27753159

[87] Yagi M Tanaka A Namisaki T Takahashi A Abe M Honda A. Is patient-reported outcome improved by nalfurafine hydrochloride in patients with primary biliary cholangitis and refractory pruritus? A post-marketing, single-arm, prospective study. J Gastroenterol. (2018) 53:1151–8. 10.1007/s00535-018-1465-z 29663077

[88] clinicaltrial.gov: Safety, Pharmacokinetics Preliminary Efficacy of Asimadoline in Pruritus Associated With Atopic Dermatitis. Study Director: Dawn McGuire, Tioga Pharmaceuticals, Inc. (2017).

[89] Cowan A Kehner GB Inan S. Targeting itch with ligands selective for κ opioid receptors. In: Cowan A Yosipovitch G editors. Pharmacology of Itch. Berlin, Heidelberg: Springer Berlin Heidelberg (2015). p. 291–314.

[90] Kremer AE van Dijk R Leckie P Schaap FG Kuiper EM Mettang T. Serum autotaxin is increased in pruritus of cholestasis, but not of other origin, and responds to therapeutic interventions. Hepatology. (2012) 56:1391–400. 10.1002/hep.25748 22473838

[91] Hashimoto T Ohata H Momose K. Itch-scratch responses induced by lysophosphatidic acid in mice. Pharmacology. (2004) 72:51–6. 10.1159/000078632 15292655

[92] Tanaka M Okudaira S Kishi Y Ohkawa R Iseki S Ota M. Autotaxin stabilizes blood vessels and is required for embryonic vasculature by producing lysophosphatidic acid. J Biol Chem. (2006) 281:25822–30. 10.1074/jbc.M605142200 16829511

[93] Tokumura A Majima E Kariya Y Tominaga K Kogure K Yasuda K. Identification of human plasma lysophospholipase D, a lysophosphatidic acid-producing enzyme, as autotaxin, a multifunctional phosphodiesterase. J Biol Chem. (2002) 277:39436–42. 10.1074/jbc.M205623200 12176993

[94] Umezu-Goto M Kishi Y Taira A Hama K Dohmae N Takio K. Autotaxin has lysophospholipase D activity leading to tumor cell growth and motility by lysophosphatidic acid production. J Cell Biol. (2002) 158:227–33. 10.1083/jcb.200204026 12119361 PMC2173129

[95] Moolenaar WH Perrakis A. Insights into autotaxin: how to produce and present a lipid mediator. Nat Rev Mol Cell Biol. (2011) 12:674–9. 10.1038/nrm3188 21915140

[96] van Meeteren LA Ruurs P Stortelers C Bouwman P van Rooijen MA Pradere JP. Autotaxin, a secreted lysophospholipase D, is essential for blood vessel formation during development. Mol Cell Biol. (2006) 26:5015–22. 10.1128/MCB.02419-05 16782887 PMC1489177

[97] Jankowski M Autotaxin: its role in biology of melanoma cells and as a pharmacological target. Enzyme Res. (2011) 2011:194857. 10.4061/2011/194857 21423677 PMC3057024

[98] Bolier R Tolenaars D Kremer AE Saris J Pares A Verheij J. Enteroendocrine cells are a potential source of serum autotaxin in men. Biochim Biophys Acta. (2016) 1862:696–704. 10.1016/j.bbadis.2016.01.012 26775031

[99] Meyer Zu Heringdorf D. Lysophospholipid receptor-dependent and -independent calcium signaling. J Cell Biochem. (2004) 92:937–48. 10.1002/jcb.20107 15258917

[100] van Meeteren LA Moolenaar WH. Regulation and biological activities of the autotaxin-LPA axis. Prog Lipid Res. (2007) 46:145–60. 10.1016/j.plipres.2007.02.001 17459484

[101] Robering JW Gebhardt L Wolf K Kuhn H Kremer AE Fischer MJM. Lysophosphatidic acid activates satellite glia cells and Schwann cells. Glia. (2019) 67:999–1012. 10.1002/glia.23585 30637823

[102] Choi JW Chun J. Lysophospholipids and their receptors in the central nervous system. Biochim Biophys Acta. (2013) 1831:20–32. 10.1016/j.bbalip.2012.07.015 22884303 PMC3693945

[103] Kittaka H Uchida K Fukuta N Tominaga M. Lysophosphatidic acid-induced itch is mediated by signalling of LPA5 receptor, phospholipase D and TRPA1/TRPV1. J Physiol. (2017) 595:2681–98. 10.1113/JP273961 28176353 PMC5390871

[104] Hausmann J Kamtekar S Christodoulou E Day JE Wu T Fulkerson Z. Structural basis of substrate discrimination and integrin binding by autotaxin. Nat Struct Mol Biol. (2011) 18:198–204. 10.1038/nsmb.1980 21240271 PMC3064516

[105] Inoue K Tanaka N Haga A Yamasaki K Umeda T Kusakabe Y. Crystallization and preliminary X-ray crystallographic analysis of human autotaxin. Acta Crystallogr Sect F Struct Biol Cryst Commun. (2011) 67:450–3. 10.1107/S174430911005311X 21505238 PMC3080147

[106] Nishimasu H Okudaira S Hama K Mihara E Dohmae N Inoue A. Crystal structure of autotaxin and insight into GPCR activation by lipid mediators. Nat Struct Mol Biol. (2011) 18:205–12. 10.1038/nsmb.1998 21240269

[107] Keune WJ Hausmann J Bolier R Tolenaars D Kremer A Heidebrecht T. Steroid binding to Autotaxin links bile salts and lysophosphatidic acid signalling. Nat Commun. (2016) 7:11248. 10.1038/ncomms11248 27075612 PMC4834639

[108] Kremer AE Le Cleac'h A Lemoinne S Wolf K De Chaisemartin L Chollet-Martin S. Antipruritic effect of bezafibrate and serum autotaxin measures in patients with primary biliary cholangitis. Gut. (2019) 68:1902–3. 10.1136/gutjnl-2018-317426 30228218

[109] Sumida H Nakamura K Yanagida K Ohkawa R Asano Y Kadono T. Decrease in circulating autotaxin by oral administration of prednisolone. Clin Chim Acta. (2013) 415:74–80. 10.1016/j.cca.2012.10.003 23063960

[110] Heerkens M Dedden S Scheepers H Van Paassen P Masclee A de Die-Smulders C. Effect of plasmapheresis on cholestatic pruritus and autotaxin activity during pregnancy. Hepatology. (2019) 69:2707–10. 10.1002/hep.30496 30614557 PMC6593664

[111] Al-Dury S Wahlstrom A Wahlin S Langedijk J Elferink RO Stahlman M. Pilot study with IBAT inhibitor A4250 for the treatment of cholestatic pruritus in primary biliary cholangitis. Sci Rep. (2018) 8:6658. 10.1038/s41598-018-25214-0 29704003 PMC5923243

[112] Hegade VS Kendrick SF Dobbins RL Miller SR Thompson D Richards D. Effect of ileal bile acid transporter inhibitor GSK2330672 on pruritus in primary biliary cholangitis: a double-blind, randomised, placebo-controlled, crossover, phase 2a study. Lancet. (2017) 389:1114–23. 10.1016/S0140-6736(17)30319-7 28187915

[113] Hegade VS Pechlivanis A McDonald JAK Rees D Corrigan M Hirschfield GM. Autotaxin, bile acid profile and effect of ileal bile acid transporter inhibition in primary biliary cholangitis patients with pruritus. Liver Int. (2019) 39:967–75. 10.1111/liv.14069 30735608

[114] Fujimori N Umemura T Kimura T Tanaka N Sugiura A Yamazaki T. Serum autotaxin levels are correlated with hepatic fibrosis and ballooning in patients with non-alcoholic fatty liver disease. World J Gastroenterol. (2018) 24:1239–49. 10.3748/wjg.v24.i11.1239 29568204 PMC5859226

[115] Joshita S Umemura T Usami Y Yamashita Y Norman GL Sugiura A. Serum autotaxin is a useful disease progression marker in patients with primary biliary cholangitis. Sci Rep. (2018) 8:8159. 10.1038/s41598-018-26531-0 29802350 PMC5970155

[116] Wunsch E Krawczyk M Milkiewicz M Trottier J Barbier O Neurath MF. Serum autotaxin is a marker of the severity of liver injury and overall survival in patients with cholestatic liver diseases. Sci Rep. (2016) 6:30847. 10.1038/srep30847 27506882 PMC4978954

[117] Joshita S Ichikawa Y Umemura T Usami Y Sugiura A Shibata S. Serum autotaxin is a useful liver fibrosis marker in patients with chronic hepatitis B virus infection. Hepatol Res. (2018) 48:275–85. 10.1111/hepr.12997 29114991

[118] Yamazaki T Joshita S Umemura T Usami Y Sugiura A Fujimori N. Association of serum autotaxin levels with liver fibrosis in patients with chronic hepatitis C. Sci Rep. (2017) 7:46705. 10.1038/srep46705 28425454 PMC5397977

[119] Fujino H Tanaka M Imamura M Morio K Ono A Nakahara T. Pruritus in patients with chronic liver disease and serum autotaxin levels in patients with primary biliary cholangitis. BMC Gastroenterol. (2019) 19:169. 10.1186/s12876-019-1092-z 31651244 PMC6813053

[120] Beck PW Handwerker HO. Bradykinin and serotonin effects on various types of cutaneous nerve fibers. Pflugers Arch. (1974) 347:209–22. 10.1007/BF00592598 4857072

[121] Akiyama T Carstens MI Carstens E. Facial injections of pruritogens and algogens excite partly overlapping populations of primary and second-order trigeminal neurons in mice. J Neurophysiol. (2010) 104:2442–50. 10.1152/jn.00563.2010 20739601 PMC3350035

[122] Klein A Carstens MI Carstens E. Facial injections of pruritogens or algogens elicit distinct behavior responses in rats and excite overlapping populations of primary sensory and trigeminal subnucleus caudalis neurons. J Neurophysiol. (2011) 106:1078–88. 10.1152/jn.00302.2011 21653727 PMC3174811

[123] Kushnir-Sukhov NM Brown JM Wu Y Kirshenbaum A Metcalfe DD. Human mast cells are capable of serotonin synthesis and release. J Allergy Clin Immunol. (2007) 119:498–9. 10.1016/j.jaci.2006.09.003 17291861

[124] McNeil B Dong X. Peripheral mechanisms of itch. Neurosci Bull. (2012) 28:100–10. 10.1007/s12264-012-1202-1 22466121 PMC5560392

[125] Zhao ZQ Liu XY Jeffry J Karunarathne WK Li JL Munanairi A. Descending control of itch transmission by the serotonergic system *via* 5-HT1A-facilitated GRP-GRPR signaling. Neuron. (2014) 84:821–34. 10.1016/j.neuron.2014.10.003 25453842 PMC4254557

[126] Schworer H Hartmann H Ramadori G. Relief of cholestatic pruritus by a novel class of drugs: 5-hydroxytryptamine type 3 (5-HT3) receptor antagonists: effectiveness of ondansetron. Pain. (1995) 61:33–7. 10.1016/0304-3959(94)00145-5 7644246

[127] Soga F Katoh N Inoue T Kishimoto S. Serotonin activates human monocytes and prevents apoptosis. J Invest Dermatol. (2007) 127:1947–55. 10.1038/sj.jid.5700824 17429435

[128] Tian B Wang XL Huang Y Chen LH Cheng RX Zhou FM. Peripheral and spinal 5-HT receptors participate in cholestatic itch and antinociception induced by bile duct ligation in rats. Sci Rep. (2016) 6:36286. 10.1038/srep36286 27824106 PMC5099756

[129] Bockaert J Claeysen S Becamel C Dumuis A Marin P. Neuronal 5-HT metabotropic receptors: fine-tuning of their structure, signaling, and roles in synaptic modulation. Cell Tissue Res. (2006) 326:553–72. 10.1007/s00441-006-0286-1 16896947

[130] Hoyer D Martin G. 5-HT receptor classification and nomenclature: towards a harmonization with the human genome. Neuropharmacology. (1997) 36:419–28. 10.1016/S0028-3908(97)00036-1 9225265

[131] Akiyama T Ivanov M Nagamine M Davoodi A Carstens MI Ikoma A. Involvement of TRPV4 in serotonin-evoked scratching. J Invest Dermatol. (2016) 136:154–60. 10.1038/JID.2015.388 26763435 PMC4731048

[132] Yamaguchi T Nagasawa T Satoh M Kuraishi Y. Itch-associated response induced by intradermal serotonin through 5-HT2 receptors in mice. Neurosci Res. (1999) 35:77–83. 10.1016/S0168-0102(99)00070-X 10616911

[133] Snyder LM Kuzirian MS Ross SE. An unexpected role for TRPV4 in serotonin-mediated itch. J Invest Dermatol. (2016) 136:7–9. 10.1016/j.jid.2015.11.010 26763416 PMC4758363

[134] Ross SE. Pain and itch: insights into the neural circuits of aversive somatosensation in health and disease. Curr Opin Neurobiol. (2011) 21:880–7. 10.1016/j.conb.2011.10.012 22054924

[135] Morita T McClain SP Batia LM Pellegrino M Wilson SR Kienzler MA. HTR7 Mediates serotonergic acute and chronic itch. Neuron. (2015) 87:124–38. 10.1016/j.neuron.2015.05.044 26074006 PMC4536073

[136] Browning J Combes B Mayo MJ. Long-term efficacy of sertraline as a treatment for cholestatic pruritus in patients with primary biliary cirrhosis. Am J Gastroenterol. (2003) 98:2736–41. 10.1111/j.1572-0241.2003.08662.x 14687826

[137] Mayo MJ Handem I Saldana S Jacobe H Getachew Y Rush AJ. Sertraline as a first-line treatment for cholestatic pruritus. Hepatology. (2007) 45:666–74. 10.1002/hep.21553 17326161

[138] Stander S Bockenholt B Schurmeyer-Horst F Weishaupt C Heuft G Luger TA. Treatment of chronic pruritus with the selective serotonin re-uptake inhibitors paroxetine and fluvoxamine: results of an open-labelled, two-arm proof-of-concept study. Acta Derm Venereol. (2009) 89:45–51. 10.2340/00015555-0553 19197541

[139] Thebaut A Habes D Gottrand F Rivet C Cohen J Debray D. Sertraline as an additional treatment for cholestatic pruritus in children. J Pediatr Gastroenterol Nutr. (2017) 64:431–5. 10.1097/MPG.0000000000001385 27557426

[140] Sommer C Serotonin in pain and analgesia: actions in the periphery. Mol Neurobiol. (2004) 30:117–25. 10.1385/MN:30:2:117 15475622

[141] European Association for the Study of the Liver. EASL clinical practice guidelines: management of cholestatic liver diseases. J Hepatol. (2009) 51:237–67. 10.1016/j.jhep.2009.04.009 19501929

[142] Gupta MA Gupta AK. Depression modulates pruritus perception. A study of pruritus in psoriasis, atopic dermatitis and chronic idiopathic urticaria. Ann NY Acad Sci. (1999) 885:394–5. 10.1111/j.1749-6632.1999.tb08697.x 10816673

[143] Gittlen SD Schulman ES Maddrey WC. Raised histamine concentrations in chronic cholestatic liver disease. Gut. (1990) 31:96–9. 10.1136/gut.31.1.96 2108078 PMC1378348

[144] Ikoma A Handwerker H Miyachi Y Schmelz M. Electrically evoked itch in humans. Pain. (2005) 113:148–54. 10.1016/j.pain.2004.10.003 15621375

[145] Jones EA Bergasa NV. Evolving concepts of the pathogenesis and treatment of the pruritus of cholestasis. Can J Gastroenterol. (2000) 14:33–40. 10.1155/2000/747495 10655025

[146] Schmelz M Schmidt R Weidner C Hilliges M Torebjork HE Handwerker HO. Chemical response pattern of different classes of C-nociceptors to pruritogens and algogens. J Neurophysiol. (2003) 89:2441–8. 10.1152/jn.01139.2002 12611975

[147] Meixiong J Vasavda C Green D Zheng Q Qi L Kwatra SG. Identification of a bilirubin receptor that may mediate a component of cholestatic itch. Elife. (2019) 8:44116. 10.7554/eLife.44116 30657454 PMC6368403

[148] Dong X Han S Zylka MJ Simon MI Anderson DJ. A diverse family of GPCRs expressed in specific subsets of nociceptive sensory neurons. Cell. (2001) 106:619–32. 10.1016/S0092-8674(01)00483-4 11551509

[149] Flegel C Schobel N Altmuller J Becker C Tannapfel A Hatt H. RNA-Seq analysis of human trigeminal and dorsal root ganglia with a focus on chemoreceptors. PLoS ONE. (2015) 10:e0128951. 10.1371/journal.pone.0128951 26070209 PMC4466559

[150] Kremer AE Gonzales E Schaap FG Oude Elferink RP Jacquemin E Beuers U. Serum autotaxin activity correlates with pruritus in pediatric cholestatic disorders. J Pediatr Gastroenterol Nutr. (2016) 62:530–5. 10.1097/MPG.0000000000001044 26628447

[151] Strassburg CP. Hyperbilirubinemia syndromes (Gilbert-Meulengracht, Crigler-Najjar, Dubin-Johnson, Rotor syndrome). Best Pract Res Clin Gastroenterol. (2010) 24:555–71. 10.1016/j.bpg.2010.07.007 20955959

[152] Reyes H Sjovall J. Bile acids and progesterone metabolites in intrahepatic cholestasis of pregnancy. Ann Med. (2000) 32:94–106. 10.3109/07853890009011758 10766400

[153] Abu-Hayyeh S Papacleovoulou G Lovgren-Sandblom A Tahir M Oduwole O Jamaludin NA. Intrahepatic cholestasis of pregnancy levels of sulfated progesterone metabolites inhibit farnesoid X receptor resulting in a cholestatic phenotype. Hepatology. (2013) 57:716–26. 10.1002/hep.26055 22961653 PMC3592994

[154] Meng LJ Reyes H Palma J Hernandez I Ribalta J Sjovall J. Profiles of bile acids and progesterone metabolites in the urine and serum of women with intrahepatic cholestasis of pregnancy. J Hepatol. (1997) 27:346–57. 10.1016/S0168-8278(97)80181-X 9288610

[155] Glantz A Reilly SJ Benthin L Lammert F Mattsson LA Marschall HU. Intrahepatic cholestasis of pregnancy: amelioration of pruritus by UDCA is associated with decreased progesterone disulphates in urine. Hepatology. (2008) 47:544–51. 10.1002/hep.21987 17968976

[156] Abu-Hayyeh S Ovadia C Lieu T Jensen DD Chambers J Dixon PH. Prognostic and mechanistic potential of progesterone sulfates in intrahepatic cholestasis of pregnancy and pruritus gravidarum. Hepatology. (2016) 63:1287–98. 10.1002/hep.28265 26426865 PMC4869673

[157] Abu-Hayyeh S Martinez-Becerra P Sheikh Abdul Kadir SH Selden C Romero MR Rees M. Inhibition of Na+-taurocholate Co-transporting polypeptide-mediated bile acid transport by cholestatic sulfated progesterone metabolites. J Biol Chem. (2010) 285:16504–12. 10.1074/jbc.M109.072140 20177056 PMC2878045

[158] Vallejo M Briz O Serrano MA Monte MJ Marin JJ. Potential role of trans-inhibition of the bile salt export pump by progesterone metabolites in the etiopathogenesis of intrahepatic cholestasis of pregnancy. J Hepatol. (2006) 44:1150–7. 10.1016/j.jhep.2005.09.017 16458994

[159] Akiyama T Lerner EA Carstens E. Protease-activated receptors and itch. Handb Exp Pharmacol. (2015) 226:219–35. 10.1007/978-3-662-44605-8_13 25861783 PMC5540334

[160] Briot A Deraison C Lacroix M Bonnart C Robin A Besson C. Kallikrein 5 induces atopic dermatitis-like lesions through PAR2-mediated thymic stromal lymphopoietin expression in Netherton syndrome. J Exp Med. (2009) 206:1135–47. 10.1084/jem.20082242 19414552 PMC2715042

[161] Kempkes C Buddenkotte J Cevikbas F Buhl T Steinhoff M. Role of PAR-2 in neuroimmune communication and itch. In: Carstens E Akiyama T editors. Itch: Mechanisms and Treatment. Boca Raton, FL: CRC Press/Taylor & Francis (2014). 24829999

[162] Steinhoff M Neisius U Ikoma A Fartasch M Heyer G Skov PS. Proteinase-activated receptor-2 mediates itch: a novel pathway for pruritus in human skin. J Neurosci. (2003) 23:6176–80. 10.1523/JNEUROSCI.23-15-06176.2003 12867500 PMC6740542

[163] Zhao J Munanairi A Liu XY Zhang J Hu L Hu M. PAR2 Mediates itch *via* TRPV3 signaling in keratinocytes. J Invest Dermatol. (2020) 140:1524–32. 10.1016/j.jid.2020.01.012 32004565 PMC7387154

[164] Ui H Andoh T Lee JB Nojima H Kuraishi Y. Potent pruritogenic action of tryptase mediated by PAR-2 receptor and its involvement in anti-pruritic effect of nafamostat mesilate in mice. Eur J Pharmacol. (2006) 530:172–8. 10.1016/j.ejphar.2005.11.021 16359660

[165] Thomsen JS Sonne M Benfeldt E Jensen SB Serup J Menne T. Experimental itch in sodium lauryl sulphate-inflamed and normal skin in humans: a randomized, double-blind, placebo-controlled study of histamine and other inducers of itch. Br J Dermatol. (2002) 146:792–800. 10.1046/j.1365-2133.2002.04722.x 12000375

[166] Shimada SG Shimada KA Collins JG. Scratching behavior in mice induced by the proteinase-activated receptor-2 agonist, SLIGRL-NH2. Eur J Pharmacol. (2006) 530:281–3. 10.1016/j.ejphar.2005.11.012 16356490

[167] Costa R Marotta DM Manjavachi MN Fernandes ES Lima-Garcia JF Paszcuk AF. Evidence for the role of neurogenic inflammation components in trypsin-elicited scratching behaviour in mice. Br J Pharmacol. (2008) 154:1094–103. 10.1038/bjp.2008.172 18454165 PMC2451046

[168] Liu Q Weng HJ Patel KN Tang Z Bai H Steinhoff M. The distinct roles of two GPCRs, MrgprC11 and PAR2, in itch and hyperalgesia. Sci Signal. (2011) 4:ra45. 10.1126/scisignal.2001925 21775281 PMC3144551

[169] Ghent CN Carruthers SG. Treatment of pruritus in primary biliary cirrhosis with rifampin. Results of a double-blind, crossover, randomized trial. Gastroenterology. (1988) 94:488–93. 10.1016/0016-5085(88)90442-8 3275568

[170] Pereira SP Rhodes JM Campbell BJ Kumar D Bain IM Murphy GM. Biliary lactoferrin concentrations are increased in active inflammatory bowel disease: a factor in the pathogenesis of primary sclerosing cholangitis? Clin Sci (Lond). (1998) 95:637–44. 10.1042/cs0950637 9791051

[171] Li Y Tang R Leung PSC Gershwin ME Ma X. Bile acids and intestinal microbiota in autoimmune cholestatic liver diseases. Autoimmun Rev. (2017) 16:885–96. 10.1016/j.autrev.2017.07.002 28698093

[172] Lv LX Fang DQ Shi D Chen DY Yan R Zhu YX. Alterations and correlations of the gut microbiome, metabolism and immunity in patients with primary biliary cirrhosis. Environ Microbiol. (2016) 18:2272–86. 10.1111/1462-2920.13401 27243236

[173] Tang R Wei Y Li Y Chen W Chen H Wang Q. Gut microbial profile is altered in primary biliary cholangitis and partially restored after UDCA therapy. Gut. (2018) 67:534–41. 10.1136/gutjnl-2016-313332 28213609

[174] Matsumoto M Aranami A Ishige A Watanabe K Benno Y. LKM512 yogurt consumption improves the intestinal environment and induces the T-helper type 1 cytokine in adult patients with intractable atopic dermatitis. Clin Exp Allergy. (2007) 37:358–70. 10.1111/j.1365-2222.2007.02642.x 17359386

[175] Matsumoto M Ebata T Hirooka J Hosoya R Inoue N Itami S. Antipruritic effects of the probiotic strain LKM512 in adults with atopic dermatitis. Ann Allergy Asthma Immunol. (2014) 113:209–16.e7. 10.1016/j.anai.2014.05.002 24893766

[176] Patel SP Vasavda C Ho B Meixiong J Dong X Kwatra SG. Cholestatic pruritus: emerging mechanisms and therapeutics. J Am Acad Dermatol. (2019) 81:1371–8. 10.1016/j.jaad.2019.04.035 31009666 PMC7825249

[177] Darragh M Chang JW Booth RJ Consedine NS. The placebo effect in inflammatory skin reactions: the influence of verbal suggestion on itch and weal size. J Psychosom Res. (2015) 78:489–94. 10.1016/j.jpsychores.2015.01.011 25649275

[178] Mayo MJ Pockros PJ Jones D Bowlus CL Levy C Patanwala I. A Randomized, controlled, phase 2 study of maralixibat in the treatment of itching associated with primary biliary cholangitis. Hepatol Commun. (2019) 3:365–81. 10.1002/hep4.1305 30859149 PMC6396374

[179] Beuers U Drug insight: mechanisms and sites of action of ursodeoxycholic acid in cholestasis. Nat Clin Pract Gastroenterol Hepatol. (2006) 3:318–28. 10.1038/ncpgasthep0521 16741551

[180] Corpechot C Carrat F Bonnand AM Poupon RE Poupon R. The effect of ursodeoxycholic acid therapy on liver fibrosis progression in primary biliary cirrhosis. Hepatology. (2000) 32:1196–9. 10.1053/jhep.2000.20240 11093724

[181] Pares A Caballeria L Rodes J Bruguera M Rodrigo L Garcia-Plaza A. Long-term effects of ursodeoxycholic acid in primary biliary cirrhosis: results of a double-blind controlled multicentric trial. UDCA-Cooperative Group from the Spanish Association for the Study of the Liver. J Hepatol. (2000) 32:561–6. 10.1016/S0168-8278(00)80216-0 10782903

[182] Pares A Cisneros L Salmeron JM Caballeria L Mas A Torras A. Extracorporeal albumin dialysis: a procedure for prolonged relief of intractable pruritus in patients with primary biliary cirrhosis. Am J Gastroenterol. (2004) 99:1105–10. 10.1111/j.1572-0241.2004.30204.x 15180733

[183] Alallam A Barth D Heathcote EJ. Role of plasmapheresis in the treatment of severe pruritus in pregnant patients with primary biliary cirrhosis: case reports. Can J Gastroenterol. (2008) 22:505–7. 10.1155/2008/969826 18478137 PMC2660806

[184] Krawczyk M Liebe R Wasilewicz M Wunsch E Raszeja-Wyszomirska J Milkiewicz P. Plasmapheresis exerts a long-lasting antipruritic effect in severe cholestatic itch. Liver Int. (2017) 37:743–7. 10.1111/liv.13281 27778443

[185] Pusl T Denk GU Parhofer KG Beuers U. Plasma separation and anion adsorption transiently relieve intractable pruritus in primary biliary cirrhosis. J Hepatol. (2006) 45:887–91. 10.1016/j.jhep.2006.08.008 17046095

[186] Verkade HJ Thompson RJ Arnell H Fischler B Gillberg PG Mattsson JP. Systematic review and meta-analysis: partial external biliary diversion in progressive familial intrahepatic cholestasis. J Pediatr Gastroenterol Nutr. (2020) 71:176–83. 10.1097/MPG.0000000000002789 32433433

[187] Barbayianni E Magrioti V Moutevelis-Minakakis P Kokotos G. Autotaxin inhibitors: a patent review. Expert Opin Ther Pat. (2013) 23:1123–32. 10.1517/13543776.2013.796364 23641951

[188] Khurana S Singh P. Rifampin is safe for treatment of pruritus due to chronic cholestasis: a meta-analysis of prospective randomized-controlled trials. Liver Int. (2006) 26:943–8. 10.1111/j.1478-3231.2006.01326.x 16953834

[189] Acocella G. Pharmacokinetics and metabolism of rifampin in humans. Rev Infect Dis. (1983) 5(Suppl. 3):S428–32. 10.1093/clinids/5.Supplement_3.S428 6356276

[190] Thebaut A Debray D Gonzales E. An update on the physiopathology and therapeutic management of cholestatic pruritus in children. Clin Res Hepatol Gastroenterol. (2018) 42:103–9. 10.1016/j.clinre.2017.08.007 29031874

[191] Wietholtz H Marschall H-U Jan S Matern S. Stimulation of bile acid 6α-hydroxylation by rifampin. J Hepatol. (1996) 24:713–8. 10.1016/S0168-8278(96)80268-6 8835747

[192] Bergasa NV Sabol SL Young WS III Kleiner DE Jones EA. Cholestasis is associated with preproenkephalin mRNA expression in the adult rat liver. Am J Physiol. (1995) 268:G346–54. 10.1152/ajpgi.1995.268.2.G346 7864131

[193] Terg R Coronel E Sorda J Munoz AE Findor J. Efficacy and safety of oral naltrexone treatment for pruritus of cholestasis, a crossover, double blind, placebo-controlled study. J Hepatol. (2002) 37:717–22. 10.1016/S0168-8278(02)00318-5 12445410

[194] Rust C Sauter GH Oswald M Buttner J Kullak-Ublick GA Paumgartner G. Effect of cholestyramine on bile acid pattern and synthesis during administration of ursodeoxycholic acid in man. Eur J Clin Invest. (2000) 30:135–9. 10.1046/j.1365-2362.2000.00606.x 10651838

[195] Carey JB Jr Williams G. Relief of the pruritus of jaundice with a bile-acid sequestering resin. JAMA. (1961) 176:432–5. 10.1001/jama.1961.03040180034008 13690773

[196] European Association for the Study of the Liver. EASL clinical practice guidelines: The diagnosis and management of patients with primary biliary cholangitis. J Hepatol. (2017) 67:145–72. 10.1016/j.jhep.2017.03.022 28427765

[197] Castagna D Budd DC Macdonald SJ Jamieson C Watson AJ. Development of autotaxin inhibitors: an overview of the patent and primary literature. J Med Chem. (2016) 59:5604–21. 10.1021/acs.jmedchem.5b01599 26745766

[198] Herr DR Chew WS Satish RL Ong WY. Pleotropic roles of autotaxin in the nervous system present opportunities for the development of novel therapeutics for neurological diseases. Mol Neurobiol. (2020) 57:372–92. 10.1007/s12035-019-01719-1 31364025

